# Successful Management of Pregnancy in a Patient With Fabry Disease Receiving Continuous Enzyme Replacement Therapy: A Case Report and Literature Review

**DOI:** 10.7759/cureus.83342

**Published:** 2025-05-02

**Authors:** Yuka Kido, Mariko Nakahara, Katsutoshi Takahashi, Saya Nagasawa, Yojiro Maruyama, Daiki Ogishima

**Affiliations:** 1 Obstetrics and Gynecology, Juntendo University Nerima Hospital, Tokyo, JPN; 2 Metabolism, Showa General Hospital, Tokyo, JPN

**Keywords:** enzyme replacement therapy, fabry disease, gestational hypertension, globotriaosylceramide, placenta previa, α-galactosidase a

## Abstract

Fabry disease is an X-linked lysosomal storage disorder characterized by deficient or reduced α-galactosidase A activity, resulting in the progressive accumulation of globotriaosylceramide in vascular endothelial cells. Although traditionally considered an X-linked recessive disorder predominantly affecting the male population, the heterozygous female population frequently develops significant clinical manifestations. Pregnancies complicated by Fabry disease are associated with an increased risk of hypertensive disorders of pregnancy and exacerbation of Fabry disease-specific symptoms. While enzyme replacement therapy and chaperone therapy have revolutionized disease management, the rarity of Fabry disease has precluded the establishment of consensus guidelines for pregnancy management. We present a case of successful maternal and perinatal management in a pregnant woman with Fabry disease who maintained α-galactosidase A therapy throughout pregnancy, accompanied by a comprehensive literature review of 21 additional cases. Our review revealed predominantly favorable outcomes with enzyme replacement therapy during pregnancy, with lower rates of proteinuria (9.1% vs. 37.2%, p = 0.01) compared to historical Fabry disease cases without enzyme replacement therapy. Common prepregnancy symptoms included proteinuria (45.5%), limb pain (40.9%), and acroparesthesia (31.8%), with symptom exacerbation during pregnancy including renal dysfunction (13.6%) and limb pain (9.1%) despite continued therapy. Pregnancy complications included hypertensive disorders (13.6%), preeclampsia (9.1%), and preterm delivery (9.1%). No congenital anomalies were reported among the newborns. Our findings suggest that enzyme replacement therapy can be safely maintained during pregnancy with careful monitoring and multidisciplinary management involving Fabry disease specialists for optimal maternal and fetal outcomes.

## Introduction

Fabry disease (FD) is a progressive metabolic disorder characterized by the pathogenic accumulation of globotriaosylceramide resulting from deficient or reduced α-galactosidase A (α-GAL) activity. The natural history of untreated FD includes progressive renal failure, cardiovascular complications, and cerebrovascular events. The prevalence of FD among newborns is reported to be one in 10,000 patients, with a higher prevalence among male newborns (one in 3,125) than in female newborns (one in 100,000) [1]. While traditionally classified as an X-linked recessive disorder primarily affecting the male population, contemporary understanding [2] recognizes that the heterozygous female population frequently develops significant clinical manifestations. Some studies [2,3] reported that pregnancies complicated by FD are associated with an increased incidence of gestational hypertension and exacerbation of disease-specific symptoms.

The advent of enzyme replacement therapy (ERT) and chaperone therapy has dramatically improved the prognosis of FD [4]. However, due to the condition's rarity, consensus guidelines for management during pregnancy and the perinatal period remain lacking. This report presents our experience with the maternal and perinatal management of a pregnant woman with FD who maintained α-GAL therapy throughout pregnancy, supplemented by a comprehensive review of the relevant literature.

## Case presentation

**Figure 1:**
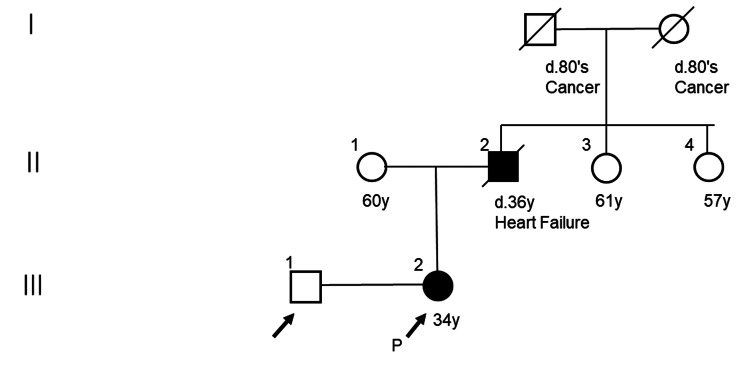
Family tree of the patient in the present case

A 37-year-old nulligravida with paternally inherited FD presented for prenatal care. Her family history was significant for her father's death from heart failure at age 36 (Figure 1).

**Figure 2:**
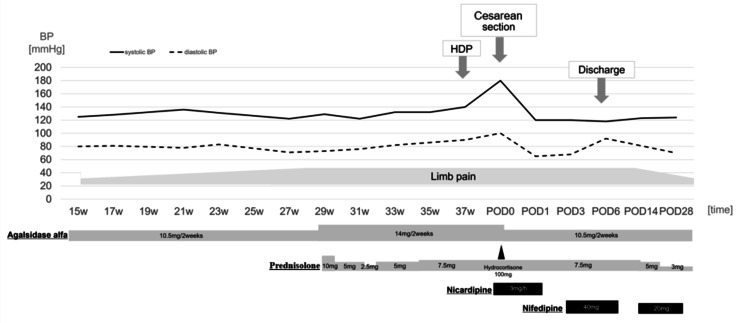
Management of Fabry disease and blood pressure (BP) during pregnancy HDP: hypertensive disorders of pregnancy; POD: postoperative day

The patient's clinical course began with corneal opacity in childhood, and reduced α-GAL activity was initially detected at age 10. Genetic testing at age 18 identified a pathogenic variant in the GLA gene (NM_000169.3:c.718_719del, p.Lys240Glu). The patient's disease progression included the onset of limb pain at age 27, leading to the initiation of ERT with α-GAL (0.2 mg/kg every 2-3 weeks) at age 30 following a pattern of remitting and relapsing symptoms. Prednisolone 3 mg/day was administered for elevated inflammatory response. The patient manifested painful subcutaneous nodules at 31 years of age. Histopathological examination of the biopsied tissue revealed non-specific findings without diagnostic features. After marriage at age 33, she pursued genetic counseling in preparation for family planning. At the age of 35, she experienced a cerebral infarction (both old and fresh lacunar infarctions), with concurrent elevation of plasma Lyso-Gb3 to 3.74 ng/mL (reference: <2 ng/mL). Following successful in vitro fertilization while maintaining ERT, the patient was referred to our institution at 14 weeks' gestation for specialized perinatal care. Initial evaluation revealed stable manifestations of FD, including chronic cerebral infarction sequelae, painful subcutaneous nodules, and urinary mulberry bodies. Cardiac function was preserved (ejection fraction 71%) with no evidence of wall motion abnormalities, diastolic dysfunction, or left ventricular hypertrophy. Renal function remained normal with no proteinuria. ERT was continued during pregnancy under multidisciplinary management, with a dose of 10.5 mg administered for patients weighing less than 55 kg. The dose was changed to 14 mg after 29 weeks' gestation when the patient's weight exceeded 55 kg. At 27 weeks' gestation, the patient manifested peripheral limb pain unresponsive to acetaminophen therapy (1,000-2,000 mg daily). Given the persistent symptoms, corticosteroid intervention was initiated with prednisolone at 10 mg daily, subsequently titrated to 7.5 mg daily according to symptom intensity. Mild gestational hypertension developed at 36 weeks' gestation but remained non-severe, with no evidence of proteinuria or end-organ damage. An elective cesarean delivery was performed at 37 weeks and one day due to partial placenta previa. Intraoperative hemorrhage from the placental separation site required intrauterine balloon tamponade for hemostasis. The procedure duration was 64 minutes, with an estimated blood loss of 1,251 g (including amniotic fluid). A male infant was delivered weighing 2,504 g (appropriate for gestational age), with Apgar scores of 8 and 9 at one and five minutes, respectively. Neonatal examination revealed no apparent congenital anomalies. Postoperative recovery was uneventful with blood pressure well-controlled on nifedipine 40 mg/day, enabling discharge on postoperative day 6. The patient's limb pain improved postpartum, allowing gradual prednisolone tapering (Figure 2).

Newborn screening for FD via expanded mass spectrometry on day 4 indicated the need for further evaluation. Subsequent testing confirmed FD diagnosis with reduced leukocyte α-GAL activity (0.1 nmol/hr/mL). Genetic testing was also performed on the infant, and the same pathogenic variants as the mother were detected, leading to a definitive diagnosis of FD.

## Discussion

This report describes the successful management of pregnancy in a woman with FD and prior cerebrovascular complications, demonstrating favorable maternal and fetal outcomes with continued ERT. Despite developing mild gestational hypertension and requiring cesarean delivery for placenta previa, the patient maintained stable disease status without stroke recurrence or cardiac dysfunction through term gestation. Current therapeutic options for FD include ERT and chaperone therapy. ERT, established approximately two decades ago, functions by supplementing agalsidase to facilitate globotriaosylceramide clearance from affected cells [5]. To contextualize our experience, we manually conducted a literature review using the search formula “fabry AND pregnancy AND enzyme replacement therapy” in the PubMed database (June 1997 to April 2024) without language or geographic restrictions. After excluding French-language publications and reviews, we identified 22 cases of FD-complicated pregnancies managed with continued ERT (including our case) (Table 1) [6-17]. A seminal study by Holmes and Laney analyzing 41 women with FD (102 pregnancies) reported ERT use in only four cases (9.8%) [3]. Comparative analysis of pregnancies with continued ERT (Group A) versus those without (Group B) revealed several notable findings (Table 2).

**Table 1:** Case series of enzyme replacement therapy during pregnancy (maternal course) FD: Fabry disease; GA: gestational age; GH: gestational hypertension; HDP: hypertensive disorders of pregnancy; NA: not available

No.	Authors, year	Age at pregnancy (years)	Age at diagnosis (years)	Variant	Fabry signs and symptoms	Drugs during pregnancy	Worsening symptoms of FD during pregnancy	Pregnancy complications
1	Wendt et al. [6], 2005	34	15	NA	Joint pain, diarrhea and cramps, proteinuria, fatigue	Agalsidase alfa	-	-
2	Kalkum et al. [7], 2009	33	30	p.Ala143Thr	Burning pain, hypohidrosis, angiokeratoma, migraine, tinnitus, depression, proteinuria	Agalsidase alfa	Pain in the legs	Gestational diabetes, preterm PROM
3	Kalkum et al. [7], 2009	38	34	p.Asn320Ile	Acroparesthesia, abdominal pain, chronic neuropathic pain, hypertension	Agalsidase alfa	Headache in the 19th week of gestation	-
4	Parent et al. [8], 2009	36	34	p.Cys56Ter (c.168C4A)	Acroparesthesia, tinnitus, hearing loss, hypohidrosis, cold intolerance, abdominal cramping, diarrhea	Agalsidase beta	-	-
5	Germain et al. [9], 2010	21	13	p.Cys52Arg	Peripheral neuropathic pains, diarrhea, hypohidrosis, edema, proteinuria	Agalsidase beta	-	-
6	Bouwman et al. [10], 2010	24	21	p.Arg310Ter	Acroparesthesia, proteinuria, multiple white matter abnormalities of brain	Agalsidase beta	-	-
7	Politei [11], 2010	37	31	p.Leu415Pro	Acroparesthesia, angiokeratomas, syncopal episodes, corneal verticillata, fatigue	Agalsidase beta	-	-
8	Senocak Tasci and Bicik [12], 2015	26	25	p.Leu275Phe	Corneal opacity, corneal verticillata, limb position sense deficit, proteinuria, stenosis of the common carotid artery	Agalsidase beta	-	-
9	Senocak Tasci and Bicik [12], 2015	29	28	p.Leu275Phe	Papules like angiokeratomas, photophobia, acroparesthesia, proteinuria, fatigue	Agalsidase beta	-	-
10	Iwafuchi et al. [13], 2017	22	22	p.Gly375Glu (c.1124G>A)	Proteinuria, macroscopic hematuria	Agalsidase alfa	-	-
11	Fernández et al. [14], 2019	36	35	p.Cys174Gly	Acroparesthesia, pain crisis, heat intolerance, hearing loss, peripheral nervous system involvement	Agalsidase alfa	-	-
12	Fernández et al. [14], 2019	38	35	p.Cys174Gly	Acroparesthesia, pain crisis, heat intolerance, decrease in glomerular filtration rate	Agalsidase alfa	-	-
13	Fernández et al. [14], 2019	24	22	p.Cys174Gly	Severe pain crisis, microalbuminuria	Agalsidase alfa	-	Eclampsia, hypertensive crisis, proteinuria at the 36th week of gestation
14	Fernández et al. [14], 2019	26	22	p.Cys174Gly	Severe pain crisis, microalbuminuria	Agalsidase alfa	-	-
15	Fernández et al. [14], 2019	19	16	p.Cys174Gly	Decrease in glomerular filtration rate	Agalsidase alfa	-	-
16	Fernández et al. [14], 2019	29	28	p.Cys174Gly	Decrease in glomerular filtration rate	Agalsidase alfa	Impaired renal function, kidney disease progression	-
17	Fernández et al. [14], 2019	22	18	p.Cys174Gly	Decrease in glomerular filtration rate	Agalsidase alfa	Impaired renal function, kidney disease progression	-
18	Madsen et al. [15], 2019	38	2	p.Gly85Asn	Ischemic stroke, hypertension, albuminuria, decrease in glomerular filtration rate, transient ischemic attack	Agalsidase beta, acetylic acid, labetalol	Blood pressure and albuminuria increased, renal function decreased in the 3rd trimester	Preeclampsia
19	Politt and Gaik [16], 2024	30	19	NA	Diarrhea, sweating, acroparesthesia, hypertrophied septum	Agalsidase alfa	-	-
20	Paydas and Akcabay [17], 2024	28-29	28	p.Asp313Tyr	Recurrent episodic abdominal pain	Agalsidase alfa	-	-
21	Paydas and Akcabay [17], 2024	30-31	28	p.Asp313Tyr	Recurrent episodic abdominal pain	Agalsidase alfa	-	-
22	Kido et al., 2025	37	10	c.178_179del	Limb pain, ischemic stroke, painful subcutaneous nodule	Agalsidase alfa, prednisolone	Pain in the legs	HDP (GH), placenta previa

**Table 2:** Pregnancy complications with and without enzyme replacement therapy (ERT) FD: Fabry disease; NA: not available *n = 85

Pregnancy complications	FD with ERT (n = 22)	Literature values in patients with FD without ERT [6] (n = 102)	Odds ratio (95% confidence interval)	p-value
Hypertensive disorders of pregnancy	3 (13.6%)	12 (10.8%)	1.18 (0.20-5.00)	0.73
Preeclampsia	2 (9.1%)	5 (4.9%)	1.93 (0.17-12.84)	0.61
Proteinuria	2 (9.1%)	38 (37.2%)	0.17 (0.02-0.76)	0.01
Premature delivery	2 (9.1%)	16 (18.8%)*	0.43 (0.04-2.11)	0.35
Eclampsia	1 (4.5%)	NA	NA	NA
Gestational diabetes	1 (4.5%)	9 (8.8%)	0.49 (0.01-3.92)	0.69
Placenta previa	1 (4.5%)	NA	NA	NA

Group A complications included hypertensive disorders of pregnancy (13.6%), preeclampsia (9.1%), proteinuria (9.1%), preterm delivery (9.1%), eclampsia (4.5%), gestational diabetes (4.5%), and placenta previa (4.5%). Group B demonstrated comparable rates of gestational hypertension (10.8%) and preeclampsia (4.9%) but higher rates of proteinuria (37.2%) [3]. Statistical analysis was performed using R software version 4.3.3 (R Foundation for Statistical Computing, Vienna, Austria). Due to the small sample size, Fisher's exact test was employed to compare complication rates between groups, with odds ratios and 95% confidence intervals calculated to quantify the associations. A p-value < 0.05 was considered statistically significant. Additional neonatal complications in Group A included hemangioma (4.5%), recurrent urinary tract infections (4.5%), and asthma (4.5%). Importantly, no low-birth-weight infants or congenital anomalies were reported among the 20 term deliveries (Table 3).

**Table 3:** Case series of enzyme replacement therapy during pregnancy (neonatal course) FD: Fabry disease; CS: cesarean section; eCS: emergency cesarean section; F: female; M: male; NA: not available; ND: normal vaginal delivery

No.	Authors, year	Delivery mode	Gestational age (weeks)	Sex	Birth weight (g)	Apgar score	FD	Others
1	Wendt et al. [6], 2005	NA	37	M	3,010	9/10	-	NA
2	Kalkum et al. [7], 2009	eCS	36	M	2,790	9/10	-	NA
3	Kalkum et al. [7], 2009	NA	Term	F	NA	NA	+	Hemangioma
4	Parent et al. [8], 2009	ND	38	F	2,885	2/10	-	-
5	Germain et al. [9], 2010	ND	38	M	3,120	10/10	-	NA
6	Bouwman et al. [10], 2010	NA	NA	F	NA	NA	+	NA
7	Politei [11], 2010	ND	38	M	3,300	9/10	+	NA
8	Senocak Tasci and Bicik [12], 2015	NA	40	F	3,100	NA	-	-
9	Senocak Tasci and Bicik [12], 2015	NA	40	F	3,400	NA	NA	-
10	Iwafuchi et al. [13], 2017	NA	40	F	2,734	8/9	+	NA
11	Fernández et al. [14], 2019	ND	37	F	3,800	9/10	-	Recurrent urinary tract infection
12	Fernández et al. [14], 2019	ND	39	M	3,190	8/10	-	NA
13	Fernández et al. [14], 2019	eCS	36	F	2,370	6/10	+	NA
14	Fernández et al. [14], 2019	CS	37	M	2,890	8/10	-	NA
15	Fernández et al. [14], 2019	ND	39	M	3,085	8-9/10	-	NA
16	Fernández et al. [14], 2019	ND	39	F	2,730	8-9/10	-	NA
17	Fernández et al. [14], 2019	ND	38	F	2,530	8-9/10	-	Asthma
18	Madsen et al. [15], 2019	CS	38	M	2,675	10/10	-	NA
19	Politt and Gaik [16], 2024	CS	40	NA	3,200	Normal	NA	NA
20	Paydas and Akcabay [17], 2024	ND	37	M	NA	9/10	-	-
21	Paydas and Akcabay [17], 2024	ND	37	M	NA	NA	Not tested	-
22	Kido et al., 2025	CS	37	M	2,504	8/9	+	-

The pathogenesis of hypertensive disorders in FD-complicated pregnancies likely involves disrupted angiogenic balance and placental dysfunction [18]. Recent histopathological studies have demonstrated extensive GL-3 accumulation across multiple placental cell types, including intermediate trophoblasts, endothelial cells, and smooth muscle cells of maternal vessels within the decidua [19]. While the transplacental distribution of supplemental enzymes remains incompletely characterized, our review suggests an increased occurrence of complications in late pregnancy despite ERT. Disease manifestations in women with FD are diverse, with acroparesthesia representing the most common initial symptom [20]. Prepregnancy symptoms in Group A included proteinuria (45.5%), limb pain (40.9%), acroparesthesia (31.8%), gastrointestinal symptoms (31.8%), renal dysfunction (22.7%), hypertension (9.1%), and cerebrovascular disease (9.1%) (Table 4).

**Table 4:** Symptom changes in Fabry disease (FD) during pregnancy *Including decreased glomerular filtration rate ERT: enzyme replacement therapy

Symptoms of FD	Before pregnancy (n = 22)	Worsening symptoms during pregnancy with ERT (n = 22)
Proteinuria	10 (45.5%)	2 (9.1%)
Limb pain	9 (40.9%)	2 (9.1%)
Acroparesthesia	7 (31.8%)	0 (0%)
Abdominal symptoms	7 (31.8%)	0 (0%)
Renal dysfunction*	5 (22.7%)	3 (13.6%)
Ear symptoms	3 (13.6%)	0 (0%)
Hypertension	2 (9.1%)	1 (4.5%)
Cerebrovascular disease	2 (9.1%)	0 (0%)

Our patient experienced worsening limb pain despite continued therapy, necessitating steroid administration. Pain management during pregnancy remains challenging due to limited therapeutic options, and the efficacy of steroid therapy was modest in our case.

Genetic counseling played a crucial role in this case, particularly given the X-linked inheritance pattern of FD. The identified GLA variant (NM_000169.3:c.718_719del, p.Lys240Glu) informed preconception counseling, which addressed inheritance patterns, recurrence risks, and therapeutic options during pregnancy. While preimplantation genetic testing remains unavailable in Japan, comprehensive genetic counseling facilitated informed decision-making regarding pregnancy continuation with ERT. Subsequent neonatal testing confirmed FD diagnosis, prompting ongoing genetic counseling. This case series has inherent limitations, including small sample size, potential selection bias, and incomplete data capture typical of observational studies. While current evidence suggests the safety of ERT during pregnancy, prospective studies with comprehensive outcome assessment are needed.

## Conclusions

This case demonstrates successful maternal and fetal outcomes in an FD-complicated pregnancy managed with continuous ERT. While our findings support the safety of maintained ERT during pregnancy, larger prospective studies are needed to establish definitive evidence-based guidelines. Currently, management decisions should involve careful shared decision-making between healthcare providers and patients, with individualized risk-benefit assessment.
